# The evaluation of food allergy knowledge and attitude in different food sectors and the effectiveness of video-based training

**DOI:** 10.3389/fnut.2025.1512845

**Published:** 2025-02-17

**Authors:** Tugce Aytulu, Beliz Su Gundogdu, Elif Yayci, Isil Ezelsoy, Betul Buyuktiryaki, Mert Veznikli, Sacide Rana Isik, Cansin Sackesen

**Affiliations:** ^1^American Hospital, Division of Nutrition and Dietetics, Istanbul, Türkiye; ^2^Koç University School of Medicine, Istanbul, Türkiye; ^3^Division of Pediatric Allergy, Koç University School of Medicine, Istanbul, Türkiye; ^4^Department of Biostatistics, Koç University School of Medicine, Istanbul, Türkiye; ^5^American Hospital, Division of Allergy, Istanbul, Türkiye

**Keywords:** attitude, effectiveness, food allergy, knowledge, nutrition, video-based training

## Abstract

**Objective:**

Food allergies are common in the food industry. The knowledge and attitude of catering staff are crucial in preventing food allergy (FA)-related reactions. The study aimed to determine the knowledge of FA and evaluate the effectiveness of video-based allergy training for catering personnel in three sectors: restaurants, schools, and hospitals.

**Methods:**

Research has been conducted on workers in three different catering industries: (i) restaurant chains, (ii) school kitchens, and (iii) hospital kitchens. The study involved administering surveys to the staff to measure their demographic data (11 questions), attitude (12 questions), and knowledge level (24 questions) about FA. After taking the pre-test, the participants who watched a 32-min video on FA were asked to fill out post-test questionnaires measuring their knowledge and attitudes.

**Results:**

At the beginning, 619 participants took the pre-test. 45.7 and 40.9% (*n* = 253) had a previous food Safety Certificate and food allergy training, respectively. Sixty-four percent (*n* = 397) received our video-based FA training. Among the participants who completed video-based training and the post-test, some knowledge and attitude items improved, but some did not, compared to the pre-test results. Knowledge levels were similar between the previously FA-trained and those untrained individuals.

**Conclusion:**

Our results show that the participants’ baseline level of knowledge and attitude for FA needs improvement. After the video training, the level of improvement in some items of knowledge and attitude was significant, but it was lower than expected. It is essential for training to not only provide accurate information but also aim to correct any known misconceptions.

## Introduction

In recent decades, food allergy (FA) has become an increasing concern (1) for families, clinicians, and policymakers around the world (2). Food allergy is the most common trigger of anaphylaxis in the community and can adversely affect people’s health and quality of life (3). In high-income countries, up to one in ten people may be affected by FA, and rates are increasing in low-income countries (4).

Families must carefully read ingredient labels of manufactured products, take care when ordering foods in restaurants, understand how to avoid cross-contact of safe foods with allergens, and incorporate strategies to avoid allergens at home, school, and other outings (5). There is a need for appropriate training and instruction for catering professionals, utilizing effective and practical tools (6). Previous studies have highlighted worrying gaps in restaurant staff’s knowledge of FA, which challenges their ability to deliver a safe meal to a food-allergic customer (7).

Many allergic reactions to food occur in commercial restaurants due to cross-contact, hidden food allergens, and restaurant staff’s lack of FA knowledge and ability to handle special requests (8). Different restaurant staff are involved in food preparation and service, and an error resulting in a food-allergic reaction can occur at any point in the process. Therefore, although managers should be well trained on FA and emergency preparedness, this instruction would ideally extend to all restaurant personnel (9). An essential factor in preventing allergic reactions caused by food allergies in restaurants is a comprehensive awareness of the knowledge, attitudes, and practices of the manager, food worker, and server regarding allergic reactions. However, the measures used in these studies have been limited concerning FA attitudes and practices (10). In this study, we aim to evaluate staff’s knowledge and attitudes toward FA across various food sectors such as restaurants, cafeterias in primary, secondary, and high schools, cafeterias in hospitals that serve the staff of the hospital as well as the patients while assessing the effectiveness of video-based training.

## Materials and methods

A set of questions was prepared based on literature research to conduct a study by the researchers (5, 10). Eleven questions were prepared to collect demographic information, sectors, and areas of expertise of employees in the food industry. Additionally, 12 questions were prepared to gage attitudes and 22 questions to assess knowledge levels about FA. Video-based training, based on the “EAACI Food Allergy and Anaphylaxis Guidelines” for managing FA in the community^3^, was developed and presented by a multidisciplinary group of two pediatric allergists, an adult allergist, a clinical dietitian, and medical school students (Table 1). The first 3 educational videos about FA definition, clinics, management, prevention, and the recognition and management of allergic reactions were eligible for all the participants. The next video training was prepared separately for schools, restaurant chains, and hospital kitchens. So, as a total, the participants had four consecutive video-based training. Ethical approval for this research project was granted by Koç University Medical School Clinical Research and Ethics Committee (2022.314.IRB1.123).

**Table 1:** Content of video-based training.

Video-based training
Modules for all categories
1. Food allergy definition, clinical findings, diagnosis, and treatment (allergic food list).
2. Food allergy prevention strategies when avoiding allergenic foods, food labeling and label reading, storing, preparing, cooking, and serving.
3. Recognizing allergic reactions, managing the allergic shock (anaphylaxis) treatment process.
Modules specific for school
4. Managing food allergies in the school cafeteria.
Modules specific for restaurant chain/hospital kitchen
5. Managing food allergies in restaurants.
Modules specific for hospital kitchen
6. Food service to allergic patients in the hospital.

### Data collection

The study was conducted in three different branches of the food industry, including one restaurant chain, one school kitchen, and two hospital kitchens from March 2023 to July 2023 in Istanbul, Türkiye. Before and after watching the educational videos, participants completed the same set of questions about FA named as pre-test and post-test via Qualtrics (Qualtrics, Provo, UT, USA)^1^. The total time spent by the volunteers watching all the videos was 32 min. Participants were allowed 2 weeks to complete watching the videos and take the post-test. When completed the post-test the participants could download training certificates.

The test gathers general information about the business/job structure, the participants, and their basic knowledge, attitudes, and behaviors regarding FA. True or false questions were asked about major food allergens and nutrition for individuals with FA. A 5-point Likert scale (ranging from strongly agree to disagree strongly) was used in questions about attitude. They were asked which methods they preferred to enhance knowledge about FA. The questions were prepared using some articles that were published on this subject (5, 10).

### Data analysis

The collected data was analyzed using SPSS version 26.0 (Armonk, NY: IBM Corp). Using the Pearson’s Chi-square and Fisher exact test, we compared percentage of answers given to questions before video-based training according to presence of previous FA training, food certificate and kitchen employees such as the manager, dietitian, cook, food engineer, waiter, and assistant staff. The McNemar-Bowker test was used to analyze the percent of knowledge and attitude scores between the pre-test and post-test. The significance level has been established at 5%.

## Results

### Demographic data of the participants

The study included 619 participants (235 females, 38%). The majority of the participants had a high school education (*n* = 462, 74.6%). Out of the total participants, 45.9% (*n* = 284) were servers, 27.3% (*n* = 169) were cooks, 13.4% (*n* = 83) were managers, 10% (*n* = 62) were stewards, and 1.8% (*n* = 11) and 1.6% (*n* = 10) were, respectively, food technicians and dietitians. (Supplementary eTable 1). Thirty-five (37.6%) out of 93 managers and food technicians reported at least one case of a FA-related allergic reaction in the past year (Supplementary eTable 1). Most of the participants (70.3%) expressed their preference for online training (Supplementary eTable 2).

### The knowledge level of food allergy

The knowledge of the 619 participants about FA is shown in Table 2. The correct answers to the knowledge questions related to the amount of food allergen, risk of mortality, and safe food preparation in the meals were 76.7, 81.6, and 72.5%, respectively, among 619 participants. After the video-based training, the percentage of the correct answers for the same questions were 81.1, 88.4, and 76.8%, respectively (*p* = 0.022, *p* = 0.031, *p* = 0.070, respectively).

**Table 2:** Questions about food allergy knowledge.

		BASELINE *N* = 619		PRE-TEST *N* = 397		POST-TEST *N* = 397		p*
		*n*	%	*n*	%	*n*	%	
Someone with a food allergy can safely eat small amounts of the foods they are allergic to.	TRUE	106	17.1	73	18.4	68	17.1	**0.022**
	**FALSE** *(Correct)*	**475**	**76.7**	**303**	**76.3**	**322**	**81.1**	
	UNDECIDED	38	6.1	21	5.3	7	1.8	
A person with a food allergy may die because of consuming the food they are allergic to.	**TRUE** *(Correct)*	**505**	**81.6**	**334**	**84.1**	**351**	**88.4**	**0.031**
	FALSE	75	12.1	42	10.6	38	9.6	
	UNDECIDED	39	6.3	21	5.3	8	2.0	
Removing an allergenic food from the meal after it is prepared makes it safe for the allergic customer.	TRUE	127	20.5	82	20.7	79	19.9	0.070
	**FALSE** *(Correct)*	**449**	**72.5**	**288**	**72.5**	**305**	**76.8**	
	UNDECIDED	43	6.9	27	6.8	13	3.3	

*Comparison of pre-test and post-test results according to McNemar-Bowker test. The meaning of bold values presents the correct answers to the survey questions.

### The knowledge about food allergens

When asked about common allergens, eggs were not recognized by 7.4%, milk by 8.1%, peanut by 9.8%, shellfish by 13.6%, hazelnut by 14.4%, sesame by 16.2%, pistachio by 17.8% and wheat by 19.9% (Table 3) in all participants (*n* = 619). Regarding the subgroup of 397 who completed the pre-and post-test, after the video-based training, the number of participants who did not recognize peanuts changed from 8.8 to 4.8% (*p* = 0.030), for shellfish from 10.6 to 5.1%, for hazelnut from 11.3 to 4.3%, for sesame from 13.6 to 5.5%, for pistachio from 14.1 to 6.8% and wheat from 17.1 to 6% significantly (*p* < 0.001 for all other items). With the highest recognition percentages, recognition of milk (*p* = 0.062) and eggs (*p* = 0.165) as allergens did not change significantly after the video-based training. The percentage of the answer choice as “undecided” was decreased in all categories. On the other hand, the percentage of participants who considered raisins, tomatoes, chocolate, and dried apricots as common allergens significantly increased after video-based training (Table 3).

**Table 3:** Questions about most common allergenic foods.

		BASELINE (*N* = 619)		PRE-TEST (*N* = 397)		POST-TEST (*N* = 397)		p*
		*n*	%	*n*	%	*n*	%	
Of the following foods, which ones do you think are significant allergens?								
Peanut *(correct)*	**TRUE**	**558**	**90.1**	**362**	**91.2**	**378**	**95.2**	**0.030**
	FALSE	36	5.8	22	5.5	14	3.5	
	UNDECIDED	25	4	13	3.3	5	1.3	
Tomatoes	TRUE	499	80.6	337	84.9	350	88.2	**0.021**
	**FALSE**	**71**	**11.5**	**37**	**9.3**	**34**	**8.6**	
	UNDECIDED	49	7.9	23	5.8	13	3.3	
Dairy products *(correct)*	**TRUE**	**569**	**91.9**	**374**	**94.3**	**384**	**96.7**	0.062
	FALSE	32	5.2	18	4.5	10	2.5	
	UNDECIDED	18	2.9	5	1.3	3	0.8	
Strawberries	TRUE	489	79.0	336	84.6	350	88.2	0.098
	**FALSE**	**66**	**10.7**	**34**	**8.6**	**32**	**8.1**	
	UNDECIDED	64	10.4	27	6.8	15	3.8	
Shellfish *(correct)*	**TRUE**	**535**	**86.4**	**355**	**89.4**	**377**	**95.0**	**<0.001**
	FALSE	39	6.3	21	5.3	15	3.8	
	UNDECIDED	45	7.3	21	5.3	5	1.3	
Dried Apricots	TRUE	379	61.2	267	67.3	313	78.8	**<0.001**
	**FALSE**	**134**	**21.6**	**79**	**19.9**	**62**	**15.6**	
	UNDECIDED	106	17.1	51	12.8	22	5.5	
Eggs *(correct)*	**TRUE**	**573**	**92.6**	**378**	**95.2**	**384**	**96.7**	0.165
	FALSE	25	4	11	2.8	10	2.5	
	UNDECIDED	21	3.4	8	2.0	3	0.8	
Chocolate	TRUE	520	84	342	86.1	366	92.2	**0.003**
	**FALSE**	**60**	**9.7**	**34**	**8.6**	**21**	**5.3**	
	UNDECIDED	39	6.3	21	5.3	10	2.5	
Raisins	TRUE	369	59.6	260	65.5	318	80.1	**<0.001**
	**FALSE**	**135**	**21.8**	**74**	**18.6**	**61**	**15.4**	
	UNDECIDED	115	18.6	63	15.9	18	4.5	
Hazelnut *(correct)*	**TRUE**	**530**	**85.6**	**352**	**88.7**	**380**	**95.7**	**<0.001**
	FALSE	41	6.6	19	4.8	13	3.3	
	UNDECIDED	48	7.8	26	6.5	4	1.0	
Sesame *(correct)*	**TRUE**	**519**	**83.8**	**343**	**86.4**	**375**	**94.5**	**<0.001**
	FALSE	56	9.1	30	7.6	16	4.0	
	UNDECIDED	44	7.1	24	6.0	6	1.5	
Wheat *(correct)*	**TRUE**	**496**	**80.1**	**329**	**82.9**	**373**	**94.0**	**<0.001**
	FALSE	73	11.8	42	10.6	20	5.0	
	UNDECIDED	50	8.1	26	6.5	4	1.0	
Pistachio *(correct)*	**TRUE**	**509**	**82.2**	**341**	**85.9**	**370**	**93.2**	**<0.001**
	FALSE	52	8.4	29	7.3	19	4.8	
	UNDECIDED	58	9.4	27	6.8	8	2.0	

*Comparison of pre-test and post-test results according to McNemar-Bowker test. The meaning of bold values presents the correct answers to the survey questions.

### The knowledge about handling and recognizing allergic reactions

96% of all participants agreed to call 911 upon a severe allergic reaction but this ratio did not increase after video-based training. Eighty-eight-point 7 % of participants believed it was appropriate to ask if the client experiencing an allergic reaction was carrying any medication. Although not significant, there was an improvement in the staff’s willingness to inquire about the person’s medication, from 89 to 93% (Table 4). Unfortunately, 37% of all participants (*n* = 619) answered the question “Suggesting the client to vomit” as a true approach and moreover in the subgroup of 397 who completed pre-test, video-based training and post-test, the percentage of the participants who think vomiting may improve the allergic reaction did not decrease (*p* = 0.97) (Table 4). When asked about allergic reaction symptoms, 66.9 and 84.2% of all participants (*n* = 619) confirmed that headache and fever were, respectively, the true symptoms of FA (Table 4). Moreover, in the subgroup who completed pre- and post-test, the ratio of the participants confirming fever and headache as a true symptom of FA reaction did not decrease. On the other hand, the ratio of the ones who correctly confirmed difficulty in breathing, rash, hives, swelling of the tongue and throat as an allergic reaction was high and improved from 90.3–91.6% to 95.5–96% significantly after the educational videos (Table 4).

**Table 4:** Questions about handling and recognizing allergic reactions.

		BASELINE (*N* = 619)		PRE-TEST (*N* = 397)		POST-TEST (*N* = 397)		p*
		*n*	%	*n*	%	*n*	%	
If a customer develops a severe allergic reaction to food, such as difficulty in breathing, which of the following should you do?								
Call 911	**TRUE *(Correct)***	**594**	**96**	**387**	**97.5**	**384**	**96.7**	**0.504**
	FALSE	12	1.9	5	1.3	10	2.5	
	UNDECIDED	13	2.1	5	1.3	3	0.8	
Asking the client if she has any medication to take with her	**TRUE *(Correct)***	**549**	**88.7**	**354**	**89.2**	**368**	**92.7**	0.215
	FALSE	48	7.8	26	6.5	19	4.8	
	UNDECIDED	22	3.6	17	4.3	10	2.5	
Suggesting the client to vomit	TRUE	229	37.0	164	41.3	169	42.6	0.097
	**FALSE *(Correct)***	**266**	**43.0**	**172**	**43.3**	**187**	**47.1**	
	UNDECIDED	124	20.0	61	15.4	41	10.3	
Which of the followings are symptoms of an allergic reaction to food?								
Difficulty in breathing	**TRUE *(Correct)***	**565**	**91.3**	**362**	**91.2**	**381**	**96.0**	**0.007**
	FALSE	31	5	21	5.3	12	3.0	
	UNDECIDED	23	3.7	14	3.5	4	1.0	
Rash or hives	**TRUE *(Correct)***	**567**	**91.6**	**372**	**93.7**	**381**	**96.0**	0.058
	FALSE	25	4	13	3.3	12	3.0	
	UNDECIDED	27	4.4	12	3.0	4	1.0	
Headache	TRUE	414	66.9	277	69.8	305	76.8	**0.002**
	**FALSE *(Correct)***	**110**	**17.8**	**73**	**18.4**	**66**	**16.6**	
	UNDECIDED	95	15.3	47	11.8	26	6.5	
Swelling of tongue and throat	**TRUE *(Correct)***	**559**	**90.3**	**363**	**91.5**	**379**	**95.5**	**0.043**
	FALSE	31	5	20	5.0	13	3.3	
	UNDECIDED	29	4.7	14	3.5	5	1.3	
Fever	TRUE	521	84.2	342	86.1	359	90.4	**0.005**
	**FALSE *(Correct)***	**40**	**6.5**	**22**	**5.5**	**27**	**6.8**	
	UNDECIDED	58	9.4	33	8.3	11	2.8	

*Comparison of pre-test and post-test results according to McNemar-Bowker test. The meaning of bold values presents the correct answers to the survey questions.

### The attitude toward food allergy

When the pre- and post-test results related to attitude toward FA were compared, the number of participants who agreed that “allergic reactions occurring in the restaurant they are working in is their responsibility” increased from 305 to 340 (*p* = 0.002). On the other hand, answers given to other attitude questions did not show significant changes with video-based training (Table 5).

**Table 5:** The comparison of attitudes for food allergy knowledge after video-based training on food allergy.

		PRE-TEST (*n* = 397)		POST-TEST (*n* = 397)		*p*
		*n*	%	*n*	%	
Service staff need to be aware of food allergies	Strongly agree	327	82.4	335	84.4	0.571
	Agree	63	15.9	58	14.6	
	Unsure	3	0.8	2	0.5	
	Disagree	2	0.5	1	0.3	
	Strongly disagree	2	0.5	1	0.3	
The kitchen staff need to be aware of food allergies	Strongly agree	321	80.9	331	83.4	0.311
	Agree	70	17.6	62	15.6	
	Unsure	4	1.0	2	0.5	
	Disagree	1	0.3	2	0.5	
	Strongly disagree	1	0.3	0	0	
Restaurants should make an effort to accommodate special requests of customers with food allergies	Strongly agree	297	74.8	311	78.3	0.360
	Agree	77	19.4	75	18.9	
	Unsure	15	3.8	8	2.0	
	Disagree	6	1.5	3	0.8	
	Strongly disagree	2	0.5	0	0	
This restaurant can accommodate special food requests from customers who have allergies.	Strongly agree	264	66.5	285	71.8	0.154
	Agree	97	24.4	89	22.4	
	Unsure	27	6.8	21	5.3	
	Disagree	5	1.3	2	0.5	
	Strongly disagree	4	1.0	0	0	
It is the customers’ responsibility to inform about their food allergies to the restaurant staff.	Strongly agree	276	69.5	274	69.0	0.556
	Agree	91	22.9	94	23.7	
	Unsure	23	5.8	19	4.8	
	Disagree	7	1.8	7	1.8	
	Strongly disagree	0	0	3	0.8	
All restaurant staff should collaborate for the demands of customers with food allergies.	Strongly agree	271	68.3	286	72.0	0.071
	Agree	99	24.9	99	24.9	
	Unsure	19	4.8	10	2.5	
	Disagree	5	1.3	2	0.5	
	Strongly disagree	3	0.8	0	0	
Allergic reactions occurring in the restaurant I am working is my responsibility.	Strongly agree	217	54.7	245	61.7	**0.002**
	Agree	88	22.2	95	23.9	
	Unsure	50	12.6	38	9.6	
	Disagree	33	8.3	15	3.8	
	Strongly disagree	9	2.3	4	1.0	

*Comparison of pre-test and post-test results according to McNemar-Bowker test. The meaning of bold values presents the correct answers to the survey questions.

### The effect of a previous food safety certificate and food allergy training on the baseline knowledge level

Among all participants (*n* = 619), 54.3 and 40.9% reported previously receiving the Food Safety Certificate and FA training (Supplementary eTable 1). When the knowledge and attitude levels of those who received food safety certification and FA training were compared with those who did not, there was no significant difference.

### Comparison of knowledge and attitude of kitchen staff according to professions and education levels

When the allergy levels were evaluated according to professions in the study, the manager, dietitian, and food technician group were significantly more successful in predicting whether wheat (*p* = 0.015) and chocolate (*p* = 0.04) are allergens than the servers, cooks, and stewards. No other significant findings regarding knowledge level were observed (Supplementary eTable 3). However the answer to the question “I prefer not to serve customers with food allergies” was statistically significant (manager vs. cook *p* < 0.001, manager vs. waiter / server *p* = 0.003, manager vs. steward *p* < 0.001) (Supplementary eTable 3).

When comparing allergy knowledge levels according to education level, those with college or higher education predicted allergens more than those with high school or less (Supplementary eTable 4).

In terms of attitude, those with high school or less agreed more with the questions “It is the customers’ responsibility to inform them about their food allergies to the restaurant staff” (*p* < 0.001), “All restaurant staff should collaborate for the demands of customers with food allergies” (*p* = 0.002), “Allergic reactions occurring in the restaurant I am working is my responsibility” (*p* < 0.001), and “The kitchen staff need to be aware of food allergies” (*p* < 0.001) (Supplementary eTable 5).

## Discussion

This study demonstrated that restaurant participants face allergic reactions in the cafeterias during their duties. The participants with a Food Safety certificate or previous FA training did not report a better knowledge and attitude level for FA, which addresses the need for a standardized program. A total of 17 questions and 13 common allergens questions about the knowledge and attitude of FA were present. The video-based training could improve only some of the items regarding knowledge and attitude. Importantly, some items related to inducing vomiting during allergic reactions, calling an emergency, identifying the wrong foods as a true allergen, and defining fever or headache as an allergic symptom did not improve. Participants in the video-based training showed a statistically significant improvement in answering questions about the most common allergenic foods. However, the rate at which foods that are not actually common allergens are marked as allergenic has increased, which shows us that we must emphasize them more clearly in video modules.

Our study noticed that while the number of undecided participants decreased, some still answered the post-test questions incorrectly. Therefore, we recommend that the video training content should include clear headings such as ‘what not to do,’ ‘non-allergic foods,’ ‘misconceptions,’ ‘things to do during treatment’ and ‘scenarios of cases’. This will help ensure that participants understand the material better and are better equipped to answer questions accurately.

Recently, Padua et al. (11) conducted a study to evaluate the efficacy of a web-based training program on improving FA management in restaurants and schools in Portugal. The authors designed FA knowledge surveys, including 20 multiple-choice questions, and prepared 9 non-interactive online training modules in video format. The surveys were applied to 216 subjects (dietitians, schoolteachers, food service workers, health students, school workers, and health professionals) before and after online training. The attitudes were not investigated in this study. The authors observed significantly increasing participants’ knowledge scores following the course (11). In our study, although most people prefer to receive information online, the results showed that online training did not significantly improve some items, which can be life-threatening. Therefore, we concluded that face-to-face and interactive training is the better approach. If this study is repeated in the future, using face-to-face training could provide more concrete evidence. However, video training can be more practical and effective in terms of being time-saving and cost-effective. In parallel with our recommendation, a previous study reported the success of a FA training event given to restaurant staff to improve the knowledge and skills for safely serving food-allergic patients (12). In that study, correct true-false answers increased from 82 to 91% before and after the face-to-face training. Also, the frequency of participants who recognized at least three common allergens increased from 9 to 64%. However, this study includes only 11 participants and needs to be further investigated in large sample sizes (12).

During our study, 86.6% of post-test participants expressed their desire for additional information related to FA. Similarly, Lee and Sozen’s research (8) found that 79.9% of the participants were interested in learning more about FA. In Bailey et al.’s study (7), 48% of the participants also expressed willingness to participate in future training.

Of 397 participants, 157 (39.5%) had received FA training before participating in our study. This contrasts with another study conducted by Sogut et al. (13) in Turkiye where only 60 (17.1%) participants reported receiving FA training. Interestingly, our study found no significant difference in the knowledge and attitude scores between those who had undergone food safety certification or FA training, and those who had not. The food safety certificate program may not include information about FA, but it is certain that previous FA training courses need revision for a better understanding and adoption to transfer the correct attitude of FA prevention and intervention of the allergic reaction to real life. Moreover, continuous training, the validity of the food and allergy certificate for a certain period of time, and its regular renewal can improve knowledge and attitude. The certificate should not be indefinite.

In our study when we examine the training subheadings of those who claimed to have received prior training (such as methods of communication, allergenic foods, and protocols), the rates of true answers fluctuate between 40 and 70%. This suggests that the quality and effectiveness of the training content should be reevaluated. Standardization is necessary in the education of the staff from each level in food preparation and service.

It seems that the rate of school cafeteria staff who have received training on FA is quite low compared to that of restaurant and hospital kitchen staff. However, these staff members serve children with common FA, so education to increase their knowledge on the subject is important for them as well as for the staff of the hospital cafeteria and restaurants.

The knowledge and attitudes were similar in different duty groups. However the answer to the question “I prefer not to serve customers with food allergies.” was significant. We also observed that as kitchen staff’s education level increases, the knowledge impoves but they are more reluctant to serve customers with allergies. The fact that those with a higher level of education (college or higher) are aware of allergies, anaphylaxis, and serious consequences may have caused them to be more reluctant in this regard. The case scenarios in the re-evaluated educational program may ameliorate this attitude.

This experience of this video-based training of food allergies in different food sectors showed that different training paths should be planned for kitchen staff at different levels and job descriptions. For example, instead of video-based training for workers who have direct responsibility in food preparation, cooking, and service, such as cooks and servers, special training such as case scenarios with allergic reactions/anaphylaxis, living labs, practical tests, and interactive training in the kitchen should be considered.

This study had several limitations. The study participants were surveyed online rather than through face-to-face interactions or workplace observation. During working hours, a group of hardworking employees were given training considering their working conditions. Post-training testing could be conducted within 2 weeks, but the long-term effectiveness of the training was indeterminable. Although we diversified the videos according to professional groups, this is insufficient. This indicates that the video content needs to be more standardized, comprehensive, or the training should be face-to-face. We think that by doing so, we can achieve much more successful results. By making the training separate and standardized according to education levels and professional groups and by identifying and addressing the deficiencies in this sector, valuable modules and standardized training can be developed not only for our country but for all countries.

In conclusion, food allergies are a common issue in the catering industry, so it is essential to improve the knowledge and attitude level of the staff regularly. Even though video training has led to significant improvements, the lower-than-expected enhancement of the staff’s knowledge and attitude in several items needs to re-evaluate the content of the training modules. Moreover, these trainings should provide not-to-do lists and correct known mistakes and case scenarios. Enriching the training content while considering the differences between sectors is essential. Expanding the video content, making it specific to professional groups, conducting it face-to-face rather than online, developing in kitchen case scenarios that are branch-specific, and making the training periodic will increase its effectiveness and benefit.

## Data Availability

The original contributions presented in the study are included in the article/Supplementary material, further inquiries can be directed to the corresponding author.

## Footnotes

^1^ https://www.qualtrics.com
