# Supplementary material for: The evaluation of food allergy knowledge and attitude in different food sectors and the effectiveness of video-based training
Source: Front Nutr. 2025 Feb 17;12:1512845. doi: 10.3389/fnut.2025.1512845 (PMC11884320; doi:10.3389/fnut.2025.1512845)
Supplement: Supplementary file 1 [file Table_1.docx]

| Supplementary Material  Supplementary eTable 1. The characteristics of the staff related with food services in the restaurants, high school and hospital | | | | | | | | | |
| --- | --- | --- | --- | --- | --- | --- | --- | --- | --- |
|  |  | **Total**  **(N = 619)** | | **Restaurant**  **(N =260)** | | **School**  **(N =63)** | | **Hospital**  **(N = 296)** | |
|  |  | **n** | % | **n** | **%** | **n** | **%** | **n** | **%** |
| Gender | Male | 384 | 62.0 | 200 | 76.9 | 25 | 39.7 | 159 | 53.7 |
|  | Female | 235 | 38.0 | 60 | 23.1 | 38 | 60.3 | 137 | 46.3 |
|  |  |  |  |  |  |  |  |  |  |
| Education level | High school diploma or less | 462 | 74.6 | 160 | 61.5 | 54 | 85.7 | 248 | 83.8 |
|  | College or higher | 157 | 25.4 | 100 | 38.5 | 9 | 14.3 | 48 | 16.2 |
|  |  |  |  |  |  |  |  |  |  |
| Duty | Manager | 83 | 13.4 | 54 | 20.8 | 5 | 7.9 | 24 | 8.1 |
|  | Dietitian | 10 | 1.6 | 2 | 0.8 | 4 | 6.3 | 4 | 1.4 |
|  | Food technician | 11 | 1.8 | 0 |  | 1 | 1.6 | 10 | 3.4 |
|  | Cook | 169 | 27.3 | 76 | 29.2 | 16 | 25.4 | 77 | 26.0 |
|  | Server | 284 | 45.9 | 116 | 44.8 | 22 | 34.9 | 146 | 49.3 |
|  | Steward | 62 | 10.0 | 12 | 4.6 | 15 | 23.8 | 35 | 11.8 |
| Previous Food Safety Certificate | Yes  No | 283  336 | 45.7  54.3 | 141  119 | 54.2  45.8 | 4  59 | 6.3  93.7 | 138  158 | 46.6  53.4 |
| Previous FA Training | Yes  No | 253  366 | 40.9  59.1 | 114  146 | 43.8  56.2 | 6  57 | 9.5  90.5 | 133  163 | 44.9  55.1 |
|  |  | **Total**  **(N =93)** | | **Restaurant**  **(N =56)** | | **School**  **(N =9)** | | **Hospital**  **(N = 28)** | |
| Number of FA related allergic reaction in the past year | 0 | 58 | 62.4 | 40 | 71.4 | 5 | 55.6 | 13 | 46.4 |
|  | 1 | 22 | 23.7 | 10 | 17.9 | 4 | 44.4 | 8 | 28.6 |
|  | 2-4 | 11 | 11.8 | 6 | 10.7 | 0 | 0 | 5 | 17.9 |
|  | ≥ 5 | 2 | 2.2 | 0 | 0 | 0 | 0 | 2 | 7.1 |

**Supplementary eTable 2.** Preferred methods by the participants to enhance the knowledge about food allergies

|  | Total  (N = 540) | | Restaurant  (N =246) | | School  (N =56) | | Hospital  (N=238) | |
| --- | --- | --- | --- | --- | --- | --- | --- | --- |
|  | **n** | **%** | **n** | **%** | **n** | **%** | **n** | **%** |
| Brochure | 83 | 15.4 | 40 | 16.3 | 3 | 0.1 | 40 | 16.8 |
| Internet | 380 | 70.3 | 188 | 76.4 | 29 | 51.8 | 163 | 50.7 |
| Face-to-face | 169 | 31.3 | 52 | 21.1 | 37 | 66.1 | 80 | 33.6 |

**Supplementary eTable 3:** Questions about most common allergenic foods by professions

|  |  | **Manager / Dietitian / Food Technician**  **n=104** | | **Cook**  **N=169** | | **Waiter/ Server**  **N=284** | | **Steward**  **N=62** | | **P** |
| --- | --- | --- | --- | --- | --- | --- | --- | --- | --- | --- |
| Of the following foods, which ones do you think are significant allergens? | | | | | | | | | | |
|  |  | **n** | **%** | **n** | **%** | **n** | **%** | **n** | **%** |  |
| Peanut  *(correct)* | **TRUE** | 98 | 94.2 | 153 | 90.5 | 251 | 88.4 | 56 | 90.3 | 0.392 |
|  | FALSE | 2 | 1.9 | 12 | 7.1 | 18 | 6.3 | 4 | 6.5 |  |
|  | UNDECIDED | 4 | 3.8 | 4 | 2.4 | 15 | 5.3 | 2 | 3.2 |  |
| Tomatoes | TRUE | 86 | 82.7 | 132 | 78.1 | 224 | 78.9 | 57 | 91.9 | 0.272 |
|  | **FALSE** | 10 | 9.6 | 23 | 13.6 | 34 | 12.0 | 4 | 6.5 |  |
|  | UNDECIDED | 8 | 7.7 | 14 | 8.3 | 26 | 9.2 | 1 | 1.6 |  |
| Dairy Products  *(correct)* | **TRUE** | 100 | 96.2 | 154 | 91.1 | 259 | 91.2 | 56 | 90.3 | 0.566 |
|  | FALSE | 3 | 2.9 | 8 | 4.7 | 16 | 5.6 | 5 | 8.1 |  |
|  | UNDECIDED | 1 | 1.0 | 7 | 4.1 | 9 | 3.2 | 1 | 1.6 |  |
| Strawberries | TRUE | 83 | 79.8 | 125 | 74.0 | 227 | 79.9 | 54 | 87.1 | 0.149 |
|  | **FALSE** | 9 | 8.7 | 26 | 15.4 | 25 | 8.8 | 6 | 9.7 |  |
|  | UNDECIDED | 12 | 11.5 | 18 | 10.7 | 32 | 11.3 | 2 | 3.2 |  |
| Shellfish  *(correct)* | **TRUE** | 94 | 90.4 | 148 | 87.6 | 241 | 84.9 | 52 | 83.9 | 0.455 |
|  | FALSE | 4 | 3.8 | 13 | 7.7 | 19 | 6.7 | 3 | 4.8 |  |
|  | UNDECIDED | 6 | 5.8 | 8 | 4.7 | 24 | 8.5 | 7 | 11.3 |  |
| Dried  Apricots | TRUE | 63 | 60.6 | 97 | 54.7 | 179 | 63.0 | 40 | 64.5 | 0.765 |
|  | **FALSE** | 24 | 23.1 | 42 | 24.9 | 54 | 19.0 | 14 | 22.6 |  |
|  | UNDECIDED | 17 | 16.2 | 30 | 17.8 | 51 | 18.0 | 8 | 12.9 |  |
| Eggs  *(correct)* | **TRUE** | 98 | 94.2 | 160 | 94.7 | 258 | 90.8 | 57 | 91.9 | 0.293 |
|  | FALSE | 3 | 2.9 | 7 | 4.7 | 11 | 3.9 | 4 | 6.5 |  |
|  | UNDECIDED | 3 | 2.9 | 2 | 1.2 | 15 | 5.3 | 1 | 1.6 |  |
| Chocolate | TRUE | 92 | 88.5 | 133 | 78.7 | 239 | 84.2 | 56 | 90.3 | 0.040* ^✦^ |
|  | **FALSE** | 8 | 7.7 | 26 | 15.4 | 21 | 7.4 | 5 | 8.1 |  |
|  | UNDECIDED | 4 | 3.8 | 10 | 5.9 | 24 | 8.5 | 1 | 1.6 |  |
| Raisins | TRUE | 61 | 58.7 | 95 | 56.2 | 173 | 60.9 | 40 | 64.5 | 0.773 |
|  | **FALSE** | 25 | 24.0 | 42 | 24.9 | 55 | 19.4 | 13 | 21.0 |  |
|  | UNDECIDED | 18 | 17.3 | 32 | 18.9 | 56 | 19.7 | 9 | 14.5 |  |
| Hazelnut  *(correct)* | **TRUE** | 95 | 91.3 | 147 | 87.0 | 236 | 83.1 | 52 | 83.9 | 0.443 |
|  | FALSE | 3 | 2.9 | 10 | 5.9 | 22 | 7.7 | 6 | 9.7 |  |
|  | UNDECIDED | 6 | 5.8 | 12 | 7.1 | 26 | 9.2 | 4 | 6.5 |  |
| Sesame  *(correct)* | **TRUE** | 94 | 90.4 | 136 | 80.5 | 239 | 84.2 | 50 | 80.6 | 0.134 |
|  | FALSE | 3 | 2.9 | 19 | 11.2 | 25 | 8.8 | 9 | 14.5 |  |
|  | UNDECIDED | 7 | 6.7 | 14 | 8.3 | 20 | 7.0 | 3 | 4.8 |  |
| Wheat  *(correct)* | **TRUE** | 91 | 87.5 | 132 | 78.1 | 229 | 80.6 | 44 | 71.0 | 0.015* ^✷^ |
|  | FALSE | 7 | 6.7 | 23 | 16.9 | 26 | 9.2 | 12 | 19.4 |  |
|  | UNDECIDED | 6 | 5.8 | 9 | 5.3 | 29 | 10.2 | 6 | 9.7 |  |
| Pistachio  *(correct)* | **TRUE** | 94 | 90.4 | 135 | 79.9 | 231 | 81.3 | 49 | 79.0 | 0.192 |
|  | FALSE | 3 | 2.9 | 18 | 10.7 | 23 ( | 8.1 | 8 | 12.9 |  |
|  | UNDECIDED | 7 | 6.7 | 16 | 9.5 | 30 | 10.6 | 5 | 8.1 |  |

^✦^ p-value <0.05 in subgroups analysis

cook vs. waiter / server = 0.020

^✷^ p-value <0.05 in subgroups analysis

cook vs. waiter / server= 0.018

manager / dietitian / food technician vs. steward = 0.026

**Supplementary eTable 3 (continued):** Questions about most common allergenic foods by professions

|  |  | **Manager / Dietitian / Food Technician**  **n=104** | | **Cook**  **N=169** | | **Waiter/ Server**  **N=284** | | **Steward**  **N=62** | | **P** |
| --- | --- | --- | --- | --- | --- | --- | --- | --- | --- | --- |
| Which of the followings are symptoms of an allergic reaction to food? | | | | | | | | | | |
|  |  | **n** | **%** | **n** | **%** | **n** | **%** | **n** | **%** |  |
| Difficulty in breathing | **TRUE *(Correct)*** | 99 | 95.2 | 153 | 90.5 | 257 | 90.5 | 56 | 90.3 | 0.290 |
|  | FALSE | 1 | 1.0 | 12 | 7.1 | 15 | 5.3 | 3 | 4.8 |  |
|  | UNDECIDED | 4 | 3.8 | 4 | 2.4 | 12 | 4.2 | 3 | 4.8 |  |
| Rash or hives | **TRUE *(Correct)*** | 102 | 98.1 | 153 | 90.5 | 255 | 89.8 | 57 | 91.9 | 0.238 |
|  | FALSE | 1 | 1.0 | 8 | 4.7 | 14 | 4.9 | 2 | 3.2 |  |
|  | UNDECIDED | 1 | 1.0 | 8 | 4.7 | 15 | 5.3 | 3 | 4.8 |  |
| Headache | TRUE | 69 | 66.3 | 109 | 64.5 | 191 | 67.3 | 45 | 72.6 | 0.328 |
|  | **FALSE *(Correct)*** | 19 | 18.3 | 35 | 20.7 | 43 | 15.1 | 13 | 21.0 |  |
|  | UNDECIDED | 16 | 15.4 | 25 | 14.8 | 50 | 17.6 | 4 | 6.5 |  |
| Swelling of tongue and throat | **TRUE *(Correct)*** | 96 | 92.3 | 153 | 90.5 | 255 | 89.8 | 55 | 88.7 | 0.717 |
|  | FALSE | 3 | 2.9 | 10 | 5.9 | 13 | 4.6 | 5 | 8.1 |  |
|  | UNDECIDED | 5 | 4.8 | 6 | 3.6 | 16 | 5.6 | 2 | 3.2 |  |
| Fever | TRUE | 85 | 81.7 | 149 | 88.2 | 241 | 84.9 | 46 | 74.2 | 0.141 |
|  | **FALSE *(Correct)*** | 6 | 5.8 | 10 | 5.9 | 16 | 5.6 | 8 | 12.9 |  |
|  | UNDECIDED | 13 | 12.5 | 10 | 5.9 | 27 | 9.5 | 8 | 12.9 |  |
| If a customer develops a severe allergic reaction to food, such as difficulty in breathing, which of the following should you do? | | | | | | | | | | |
|  |  | **n** | **%** | **n** | **%** | **n** | **%** | **n** | **%** |  |
| Call 911 | **TRUE *(Correct)*** | 100 | 96.2 | 165 | 97.6 | 268 | 94.4 | 61 | 98.4 | 0.716 |
|  | FALSE | 2 | 1.9 | 2 | 1.2 | 7 | 2.5 | 1 | 1.6 |  |
|  | UNDECIDED | 2 | 1.9 | 2 | 1.2 | 9 | 3.2 | 0 | 0 |  |
| Asking the client if she has any medication to take with her | **TRUE *(Correct)*** | 96 | 92.3 | 150 | 88.8 | 246 | 86.6 | 57 | 91.9 | 0.383 |
|  | FALSE | 3 | 2.9 | 14 | 8.3 | 27 | 9.5 | 4 | 6.5 |  |
|  | UNDECIDED | 5 | 4.8 | 5 | 3.0 | 11 | 3.9 | 1 | 1.6 |  |
| Suggesting the client to vomit | TRUE | 33 | 31.7 | 60 | 35.5 | 109 | 38.4 | 27 | 43.5 | 0.204 |
|  | **FALSE *(Correct)*** | 43 | 41.3 | 70 | 41.4 | 125 | 44.0 | 28 | 45.2 |  |
|  | UNDECIDED | 28 | 26.9 | 39 | 23.1 | 50 | 17.6 | 7 | 11.3 |  |
|  |  |  |  |  |  |  |  |  |  |  |
| Someone with a food allergy can safely eat small amounts of the foods they are allergic to. | TRUE | 13 | 12.5 | 30 | 17.8 | 46 | 16.2 | 17 | 27.4 | 0.329 |
|  | **FALSE *(Correct)*** | 83 | 79.8 | 130 | 76.9 | 221 | 77.8 | 41 | 66.1 |  |
|  | UNDECIDED | 8 | 7.7 | 9 | 5.3 | 17 | 6.0 | 4 | 6.5 |  |
| A person with a food allergy may die because of consuming the food they are allergic to. | **TRUE** ***(Correct)*** | 88 | 84.6 | 132 | 78.1 | 234 | 82.4 | 51 | 82.3 | 0.685 |
|  | FALSE | 11 | 10.6 | 25 | 14.8 | 30 | 10.6 | 9 | 14.5 |  |
|  | UNDECIDED | 5 | 4.8 | 12 | 7.1 | 20 | 7.0 | 2 | 3.2 |  |
| Removing an allergenic food from the meal after it is prepared makes it safe for the allergic customer. | TRUE | 17 | 16.3 | 30 | 17.8 | 62 | 21.8 | 18 | 29.0 | 0.489 |
|  | **FALSE** ***(Correct)*** | 80 | 76.9 | 127 | 75.1 | 203 | 71.5 | 39 | 62.9 |  |
|  | UNDECIDED | 7 | 6.7 | 12 | 7.1 | 19 | 6.7 | 5 | 8.1 |  |
| I prefer not to serve customers with food allergies. | Definitely I agree | 21 | 20.2 | 62 | 36.7 | 79 | 27.8 | 27 | 43.5 | **<0.001* ^✦^** |
|  | I agree | 7 | 6.7 | 26 | 15.4 | 43 | 15.1 | 9 | 14.5 |  |
|  | neither agree nor disagree | 10 | 9.6 | 13 | 7.7 | 28 | 9.9 | 7 | 11.3 |  |
|  | I disagree | 29 | 27.9 | 45 | 26.6 | 83 | 29.2 | 14 | 22.6 |  |
|  | Definitely I disagree | 37 | 35.6 | 23 | 13.6 | 51 | 18.0 | 5 | 8.1 |  |

^✦^ p-value <0.05 in subgroups analysis

manager vs. cook = <0.001

manager vs. waiter / server = 0.003

manager vs. steward = <0.001

**Supplementary eTable 4: Questions about most common allergenic foods by education level**

|  |  | **High school diploma or less(n=462)** | | **College or higher**  **(n=157)** | | **P** |
| --- | --- | --- | --- | --- | --- | --- |
| Which of the followings are symptoms of an allergic reaction to food? | | | | | | |
|  |  | **n** | **%** | **n** | **%** |  |
| Peanut​ | **TRUE** | 409 | 88.5 | 149 | 94.9 | 0.068 |
|  | FALSE | 31 | 6.7 | 5 | 3.2 |  |
|  | UNDECIDED | 22 | 4.8 | 3 | 1.9 |  |
| Tomatoes | TRUE | 374 | 81.0 | 125 | 79.6 | 0.665 |
|  | **FALSE** | 54 | 11.7 | 17 | 10.8 |  |
|  | UNDECIDED | 34 | 7.4 | 15 | 9.6 |  |
| Milk And milk products | **TRUE** | 416 | 90.0 | 153 | 97.5 | **0.009*** |
|  | FALSE | 29 | 6.3 | 3 | 1.9 |  |
|  | UNDECIDED | 17 | 3.7 | 1 | 0.6 |  |
| Strawberry | TRUE | 359 | 77.7 | 130 | 82.8 | 0.315 |
|  | **FALSE** | 54 | 11.7 | 12 | 7.6 |  |
|  | UNDECIDED | 49 | 10.6 | 15 | 9.6 |  |
| Shelled sea products | **TRUE** | 390 | 84.4 | 145 | 92.4 | **0.031*** |
|  | FALSE | 35 | 7.6 | 4 | 2.5 |  |
|  | UNDECIDED | 37 | 8.0 | 8 | 5.1 |  |
| Dried Apricots | TRUE | 293 | 63.4 | 86 | 54.8 | **0.038*** |
|  | **FALSE** | 100 | 21.6 | 34 | 21.7 |  |
|  | UNDECIDED | 69 | 14.9 | 37 | 23.6 |  |
| Egg | **TRUE** | 422 | 91.3 | 151 | 96.2 | 0.094 |
|  | FALSE | 23 | 5.0 | 2 | 1.3 |  |
|  | UNDECIDED | 17 | 3.7 | 4 | 2.5 |  |
| Chocolate | TRUE | 378 | 81.8 | 142 | 90.4 | **0.034*** |
|  | **FALSE** | 52 | 11.3 | 8 | 5.1 |  |
|  | UNDECIDED | 32 | 6.9 | 7 | 4.5 |  |
| Raisins​ | TRUE | 280 | 60.6 | 89 | 56.7 | 0.607 |
|  | **FALSE** | 100 | 21.6 | 35 | 22.3 |  |
|  | UNDECIDED | 82 | 17.7 | 33 | 21.0 |  |
| Hazelnut | **TRUE** | 386 | 83.5 | 144 | 91.7 | **0.014*** |
|  | FALSE | 38 | 8.2 | 3 | 1.9 |  |
|  | UNDECIDED | 38 | 8.2 | 10 | 6.4 |  |
| Sesame | **TRUE** | 377 | 81.6 | 142 | 90.4 | **0.010*** |
|  | FALSE | 51 | 11.0 | 5 | 3.2 |  |
|  | UNDECIDED | 34 | 7.4 | 10 | 6.4 |  |
| Wheat | **TRUE** | 360 | 77.9 | 136 | 86.6 | 0.051 |
|  | FALSE | 62 | 13.4 | 11 | 7.0 |  |
|  | UNDECIDED | 40 | 8.7 | 10 | 6.4 |  |
| Pistachio​ | **TRUE** | 373 | 80.7 | 136 | 86.6 | 0.229 |
|  | FALSE | 43 | 9.3 | 9 | 5.7 |  |
|  | UNDECIDED | 46 | 10.0 | 12 | 7.6 |  |

**Supplementary eTable 4 (continued): Questions about most common allergenic foods by education level**

|  |  | **High school diploma or less(n=462)** | | **College or higher**  **(n=157)** | | **P** |
| --- | --- | --- | --- | --- | --- | --- |
| Which of the followings are symptoms of an allergic reaction to food? | | | | | | |
|  |  | **n** | **%** | **n** | **%** |  |
| Difficulty in breathing | **TRUE *(Correct)*** | 414 | 89.6 | 151 | 96.2 | **0.039*** |
|  | FALSE | 27 | 5.8 | 4 | 2.5 |  |
|  | UNDECIDED | 21 | 4.5 | 2 | 1.3 |  |
| Rash or hives | **TRUE *(Correct)*** | 411 | 89.0 | 156 | 99.4 | **<0.001*** |
|  | FALSE | 25 | 5.4 | 0 | 0 |  |
|  | UNDECIDED | 26 | 5.6 | 1 | 0.6 |  |
| Headache | TRUE | 307 | 66.5 | 107 | 68.2 | 0.894 |
|  | **FALSE *(Correct)*** | 84 | 18.2 | 26 | 16.6 |  |
|  | UNDECIDED | 71 | 15.4 | 24 | 15.3 |  |
| Swelling of tongue and throat | **TRUE *(Correct)*** | 409 | 88.5 | 150 | 95.5 | **0.023*** |
|  | FALSE | 29 | 6.3 | 2 | 1.3 |  |
|  | UNDECIDED | 24 | 5.2 | 5 | 3.2 |  |
| Fever | TRUE | 381 | 82.5 | 140 | 89.2 | 0.091 |
|  | **FALSE *(Correct)*** | 35 | 7.6 | 5 | 3.2 |  |
|  | UNDECIDED | 46 | 10.0 | 12 | 7.6 |  |
| If a customer develops a severe allergic reaction to food, such as difficulty in breathing, which of the following should you do? | | | | | | |
| Call 911 | **TRUE *(Correct)*** | 440 | 95.2 | 154 | 98.1 | 0.342 |
|  | FALSE | 11 | 2.4 | 1 | 0.6 |  |
|  | UNDECIDED | 11 | 2.4 | 2 | 1.3 |  |
| Asking the client if she has any medication to take with her | **TRUE *(Correct)*** | 402 | 87.0 | 147 | 93.6 | 0.068 |
|  | FALSE | 42 | 9.1 | 6 | 3.8 |  |
|  | UNDECIDED | 18 | 3.9 | 4 | 2.5 |  |
| Suggesting the client to vomit | TRUE | 181 | 39.2 | 48 | 30.6 | 0.153 |
|  | **FALSE *(Correct)*** | 191 | 41.3 | 75 | 47.8 |  |
|  | UNDECIDED | 90 | 19.5 | 34 | 21.7 |  |
|  |  |  |  |  |  |  |
| Someone with a food allergy can safely eat small amounts of the foods they are allergic to. | TRUE | 90 | 19.5 | 16 | 10.2 | **0.012*** |
|  | **FALSE *(Correct)*** | 341 | 73.8 | 134 | 85.4 |  |
|  | UNDECIDED | 31 | 6.7 | 7 | 4.5 |  |
| A person with a food allergy may die because of consuming the food they are allergic to. | **TRUE** ***(Correct)*** | 365 | 79.0 | 140 | 89.2 | **0.008*** |
|  | FALSE | 61 | 13.2 | 14 | 8.9 |  |
|  | UNDECIDED | 36 | 7.8 | 3 | 1.9 |  |
| Removing an allergenic food from the meal after it is prepared makes it safe for the allergic customer. | TRUE | 110 | 23.8 | 17 | 10.8 | **<0.001*** |
|  | **FALSE** ***(Correct)*** | 317 | 68.6 | 132 | 84.1 |  |
|  | UNDECIDED | 35 | 7.6 | 8 | 5.1 |  |

**Supplementary eTable 5: Questions about most common allergenic foods by education level**

|  |  | **High school diploma or less(n=462)** | | **College or higher**  **(n=157)** | | **p** |
| --- | --- | --- | --- | --- | --- | --- |
|  |  | **n** | **%** | **n** | **%** |  |
| Service staff need to be aware of food allergies | Strongly agree | 380 | 82.3 | 138 | 87.9 | 0.274 |
|  | Agree | 72 | 15.6 | 15 | 9.6 |  |
|  | Unsure | 3 | 0.6 | 2 | 1.3 |  |
|  | Disagree | 4 | 0.9 | 1 | 0.6 |  |
|  | Strongly disagree | 3 | 0.6 | 1 | 0.6 |  |
| The kitchen staff need to be aware of food allergies | Strongly agree | 371 | 80.3 | 140 | 89.2 | 0.081 |
|  | Agree | 78 | 16.9 | 15 | 9.6 |  |
|  | Unsure | 10 | 2.2 | 1 | 0.6 |  |
|  | Disagree | 1 | 0.2 | 0 | 0 |  |
|  | Strongly disagree | 2 | 0.4 | 1 | 0.6 |  |
| Restaurants should make an effort to accommodate special requests of customers with food allergies | Strongly agree | 341 | 73.8 | 120 | 76.4 | 0.980 |
|  | Agree | 92 | 19.9 | 29 | 18.5 |  |
|  | Unsure | 18 | 3.9 | 5 | 3.2 |  |
|  | Disagree | 8 | 1.7 | 2 | 1.3 |  |
|  | Strongly disagree | 3 | 0.6 | 1 | 0.6 |  |
| This restaurant can accommodate special food requests from customers who have allergies. | Strongly agree | 306 | 66.2 | 104 | 66.2 | 0.158 |
|  | Agree | 115 | 24.9 | 33 | 21.0 |  |
|  | Unsure | 27 | 5.8 | 17 | 10.8 |  |
|  | Disagree | 10 | 2.2 | 1 | 0.6 |  |
|  | Strongly disagree | 4 | 0.9 | 2 | 1.3 |  |
| It is the customers' responsibility to inform about their food allergies to the restaurant staff. | Strongly agree | 297 | 64.3 | 86 | 54.8 | **<0.001*** |
|  | Agree | 124 | 26.8 | 36 | 22.9 |  |
|  | Unsure | 28 | 6.1 | 22 | 14.0 |  |
|  | Disagree | 12 | 2.6 | 10 | 6.4 |  |
|  | Strongly disagree | 1 | 0.2 | 3 | 1.9 |  |
| All restaurant staff should collaborate for the demands of customers with food allergies. | Strongly agree | 253 | 54.8 | 66 | 42.0 | **0.002*** |
|  | Agree | 107 | 23.2 | 34 | 21.7 |  |
|  | Unsure | 53 | 11.5 | 32 | 20.4 |  |
|  | Disagree | 42 | 9.1 | 17 | 10.8 |  |
|  | Strongly disagree | 7 | 1.5 | 8 | 5.1 |  |
| Allergic reactions occurring in the restaurant I am working is my responsibility. | Strongly Agree | 169 | 36.6 | 35 | 22.3 | **<0.001*** |
|  | Agree | 84 | 18.2 | 22 | 14.0 |  |
|  | Unsure | 84 | 18.2 | 24 | 15.3 |  |
|  | Disagree | 88 | 19.0 | 57 | 36.3 |  |
|  | Strongly disagree | 37 | 8.0 | 19 | 12.1 |  |
| Service staff need to be aware of food allergies | Strongly agree | 312 | 67.5 | 119 | 75.8 | 0.092 |
|  | Agree | 109 | 23.6 | 30 | 19.1 |  |
|  | Unsure | 27 | 5.8 | 8 | 5.1 |  |
|  | Disagree | 13 | 2.8 | 0 | 0 |  |
|  | Strongly disagree | 1 | 0.2 | 0 | 0 |  |
| The kitchen staff need to be aware of food allergies | Strongly agree | 162 | 35.1 | 27 | 17.2 | **<0.001*** |
|  | Agree | 75 | 16.2 | 10 | 6.4 |  |
|  | Unsure | 45 | 9.7 | 13 | 8.3 |  |
|  | Disagree | 110 | 23.8 | 61 | 38.9 |  |
|  | Strongly disagree | 70 | 15.2 | 46 | 29.3 |  |
| Restaurants should make an effort to accommodate special requests of customers with food allergies | Strongly agree | 318 | 68.8 | 109 | 69.4 | 0.319 |
|  | Agree | 113 | 24.5 | 36 | 22.9 |  |
|  | Unsure | 20 | 4.3 | 11 | 7.0 |  |
|  | Disagree | 8 | 1.7 | 0 | 0 |  |
|  | Strongly disagree | 3 | 0.6 | 1 | 0.6 |  |
